# Self-efficacy in nutritional care for older adults among hospital-based nurses: a latent profile and network simulation analysis

**DOI:** 10.1186/s12912-026-04391-8

**Published:** 2026-02-12

**Authors:** Lan-Zhi Wei, Mei-Chan Chong, Xing-Xing Lu, Nadeeka Shayamalie Gunarathne, Shu-Wen Ren

**Affiliations:** 1 Department of Nursing Science, Faculty of Medicine, Universiti Malaya, Kuala Lumpur, Malaysia; 2 Zhenjiang First People’s Hospital, Zhenjiang, 212000 China; 3 Medical College of Jiangsu University, Zhenjiang, China

**Keywords:** Nurse, Self-efficacy, Nutrition, Latent profile analysis, Computer-simulated network analysis

## Abstract

**Background:**

Malnutrition among older adults remains a critical concern. Nurses play a key role in its prevention, yet their self-efficacy in nutritional care is often overlooked.

**Purpose:**

To identify classes of self-efficacy in nutritional care for older adults among nurses and determine key intervention targets for each class.

**Method:**

A cross-sectional online survey was conducted between August and October 2024 using convenience sampling. A total of 510 nurses from different hospital grades in Jiangsu Province, China, completed a general information questionnaire and the Self-Efficacy Scale for Nursing Nutrition Care (SE-NNC). Latent profile analysis identified classes of self-efficacy, and two types of simulated interventions (weakened and enhanced) were conducted to explore key targets within each class.

**Results:**

Three classes of SE-NNC were identified: low (15.9%), moderate (49.2%), and high (34.9%). Detecting early signs of nutritional changes in older adults and interpreting anthropometric and muscle condition indices were key intervention targets in the overall population. Organizing mealtimes to facilitate complete intake and supporting older adults with personalized meal interventions were the most critical targets in the low and moderate SE-NNC, respectively. Evaluating adherence to nutrition recommendations and detecting early nutritional changes were the main focus areas in the high SE-NNC.

**Conclusion:**

Three distinct SE-NNC patterns were identified, with five key traits representing potential intervention targets for improving the self-efficacy of nurses in providing nutrition care for older adults.

**Clinical trial number:**

Not applicable.

**Supplementary Information:**

The online version contains supplementary material available at 10.1186/s12912-026-04391-8.

## Introduction

With the global population aging rapidly, malnutrition among older adults has become a serious public health concern [1]. The prevalence of malnutrition is particularly high among hospitalized older adults, reaching 22% internationally, compared to 17.5% in nursing homes and 8.7% among those receiving home care [2, 3]. In China, approximately 18.9% of hospitalized older adults, 14.1% in nursing homes, and 12.6% of those receiving home care are affected [4, 5]. This higher prevalence is partly due to hospitalization itself, which can worsen nutritional decline through acute illness, inadequate hospital meal services, and under-recognition of malnutrition, further highlighting the urgent need for prioritized nutritional assessment and intervention for hospitalized older adults [6].

As healthcare professionals providing round-the-clock care, nurses play a vital role in preventing and managing malnutrition among older adults [7]. Their responsibilities include conducting nutritional assessments, monitoring dietary intake, and communicating with patients and their families [8, 9]. However, many nurses often regard nutritional care as a secondary task. They may assume that nutritional assessment and intervention are the responsibilities of physicians or dietitians, while providing meals and assisting with feeding should be handled by family members or caregivers [10]. This negative attitude can increase the risk of malnutrition in older patients [10]. In China, although some specialized nursing fields have been established, nutrition nursing is still in its early stages [11]. The training system for nutrition specialist nurses is not yet fully developed, and the number of specialists remains limited. As a result, most nutrition care responsibilities are currently undertaken part-time by clinical nurses within their respective departments [11].

Research has shown that nurses’ self-efficacy in providing nutritional care for older adults is a key psychological mechanism influencing their care attitudes and behaviors. It reflects nurses’ perceived ability to deliver high-quality nutritional care [10]. However, most current studies evaluate self-efficacy using only total scores, which overlooks differences in individual response patterns [10]. Nurses with identical total scores may still vary significantly on specific items, and this ‘one-size-fits-all’ approach may reduce the effectiveness and precision of interventions [12]. Latent Profile Analysis (LPA) can identify potential heterogeneous classes of self-efficacy. This enables more targeted intervention strategies tailored to the characteristics of different groups, which may ultimately improve the quality of nutritional care [12].

After identifying different latent classes, it is important to understand their internal structure and core characteristics. Traditional latent variable models assume that latent factors fully explain the observed indicators, treating these indicators as mutually independent [13]. This assumption ignores potential interactions among the items. Nurses’ self-efficacy in providing nutritional care comprises three traits: nutrition care knowledge, assessment evidence and utilization, and care delivery. These traits may involve interrelationships among their respective items [14]. Network analysis can reveal these interrelationships and identify core traits within each class, providing a foundation for more targeted interventions [15].

However, network analysis is essentially a static modeling approach. It remains uncertain whether the identified “central nodes” are truly suitable as intervention targets [16]. Moreover, network-based interventions can be resource-intensive, and their limited effectiveness may delay the adoption of more efficient alternative strategies [17]. To address this, computer-simulated interventions provide a dynamic evaluation framework. By systematically adjusting the activation probabilities of nodes, these simulations model how changes in individual factors affect the overall network structure. This approach can help explore potentially important intervention targets and improve intervention efficiency [16, 17].

Therefore, this study integrates LPA with computer-simulated network interventions to: 1) identify latent classes of self-efficacy in nutritional care for older adults among nurses; 2) characterize the internal structure and core elements of each class through network analysis; and 3) determine potential intervention targets using dynamic network simulation. This integrated approach aims to provide precise theoretical insights and practical guidance for enhancing nurses’ self-efficacy in nutritional care.

## Methods

### Design

This cross-sectional study used an online survey with a convenience sampling method, targeting nurses from different hospital grades in Jiangsu Province, China, including secondary B, secondary A, tertiary B, and tertiary A hospitals. In China, hospital grades reflect technical capabilities, service capacity, and management quality, with higher-level hospitals having stronger technical and managerial resources.

### Participants and procedures

Participants were nurses from various hospitals who were directly involved in clinical care. It should be noted that professional titles (nurse, senior nurse, supervisor nurse, associate chief nurse or above) do not necessarily indicate administrative roles. The inclusion criteria were: 1) having worked in the hospital for at least 6 months [18], and 2) holding a registered nurse qualification. The exclusion criteria were: 1) nurses on sick leave or not on duty during the data collection period, and 2) nurses in administrative or logistical support positions, including managers not directly involved in clinical care and nurses from units not directly related to nutritional care, such as operating rooms, pediatrics, obstetrics, and emergency departments.

Previous studies indicate that at least 300 participants are needed to attain 80% accuracy in LPA [19, 20]. For network analysis, the sample size should be larger than the total number of estimated parameters, which include threshold parameters and pairwise correlation parameters [21]. Threshold parameters correspond to the number of nodes, and pairwise correlation parameters are calculated as P(*p*-1)/2, where P denotes the number of nodes in the partial correlation network [21, 22]. In this study, P was 27 nutritional care self-efficacy traits (nodes), resulting in a minimum required sample size of 378 participants.

Data were collected from August to October 2024. During this period, an online questionnaire was distributed to all nursing staff through their respective nursing departments. The questionnaire outlined the study objectives and informed consent requirements and was hosted on the online survey platform “Questionnaire Star.” A total of 530 questionnaires were initially distributed. To ensure data quality, all questions were mandatory, and each IP address could submit the questionnaire only once [12]. Data validation was conducted independently by two researchers. Questionnaires were excluded if they met pre-specified quality criteria: completed in less than 3 minutes (*n* = 9), containing obvious logical errors (*n* = 5), or exhibiting a high degree of response regularity (*n* = 6) [12]. After these exclusions, 510 valid questionnaires were retained, resulting in an effective response rate of 96.2%.

### Measures

#### Sociodemographic characteristics of participants

Guided by previous studies [10, 11], a range of sociodemographic and professional variables were included in the personal information form. These included, but were not limited to, gender, age (in years), marital status, years of work experience, professional title, status as a nutrition specialist nurse, and prior training in nutritional care for older adults.

#### Self-efficacy scale for nursing nutrition care (SE-NNC)

The SE-NNC was originally developed by Italian scholars as a valid and reliable tool for assessing nurses’ self-efficacy in nutritional care for older adults. It has broad applicability in clinical settings [14]. The scale consists of 27 items across three dimensions: nutritional care knowledge (6 items), assessment and evidence utilization (15 items), and care delivery (6 items). Each item is rated on a 5-point Likert scale from 1 (completely not confident) to 5 (completely confident), yielding a total score of 27–135. Higher scores indicate stronger self-efficacy in nutritional care.

The SE-NNC was translated into Chinese and validated by Deng [23]. The results showed good reliability and validity among nurses, supporting its use for evaluating the self-efficacy of nurses providing nutritional care to hospitalized older adults. In the present study, Cronbach’s alpha for the SE-NNC was 0.937.

### Data analysis

Statistical analyses were performed using SPSS version 26.0 and R version 4.2.1. First, descriptive statistics were used to summarize baseline characteristics, with continuous variables presented as means and standard deviations (as the data in this study followed a normal distribution), and categorical variables as frequencies and proportions.

Second, latent profile model fit was evaluated using Akaike Information Criterion (AIC), Bayesian Information Criterion (BIC), sample-size adjusted BIC (aBIC), Lo-Mendell-Rubin Likelihood Ratio Test (LMR-LRT), Bootstrapped Likelihood Ratio Test (BLRT), and entropy. Lower AIC, BIC, and aBIC values indicate better model fit; entropy values closer to 1.0 suggest greater classification accuracy. Significant p-values (*p* < 0.05) from LMR-LRT and BLRT support the k-class model over the k–1 class model. In addition, research interpretability was also taken into account [12].

Then, core traits within the networks were determined through centrality indices (strength, expected influence, closeness, and betweenness), with a particular focus on strength and expected influence given that closeness and betweenness are less sensitive in psychometrics [22]. The accuracy of edge weights was evaluated using non-parametric bootstrapping with 1000 samples to construct 95% confidence intervals (CIs), with wider CIs indicating lower precision and narrower CIs indicating higher reliability in edge estimation [22]. The stability of node centrality metrics (strength and expected influence) was subsequently assessed using the correlation stability coefficient (CS-C) through a case-dropping bootstrap procedure with 1000 iterations [22]. CS-C > 0.5 indicate strong stability, 0.25–0.50 acceptable stability, and < 0.25 poor stability [22].

Finally, computer-simulated interventions were performed using the NodeIdentifyR algorithm (NIRA) to identify key targets in the network. Two types of interventions were simulated: alleviating intervention and aggravating intervention [16, 24]. Given the characteristics of self-efficacy and based on guidance from previous research, the terms “alleviating intervention” and “aggravating intervention” are referred to as “simulated weakening” and “simulated enhancing,” respectively, in this study. These terms are intended to clarify our discussion and avoid any potential confusion about the nature of the work, as this study does not involve any real interventions [16]. NIRA evaluates changes in total network scores before and after simulated perturbations, and nodes associated with larger absolute score changes are interpreted as relatively influential within the simulated Ising network context [16].

As the original SE-NNC scale employed a 5-point response format, it was dichotomized into a 2-point scale to fit the requirements of the Ising model, which may have influenced the observed inter-node connections. Therefore, Gaussian Graph Models (GGM) based on continuous variables were fitted using the original scores to validate the stability of the Ising model results. All methodological specifics are outlined in Supplementary Material 1.

## Results

### Descriptive statistics

The study included a total of 33 (6.5%) males and 477 (93.5%) females, with average age was 34.21 years (SD = 7.65). A total of 423 nurses (82.9%) had bachelor’s degree. The vast majority of nurses (73.9%), were married. Additional demographic information is provided in Table 1. The mean SE-NNC score was 90.12 ± 23.38. Table 2 shows the detailed scores on SE-NNC.

**Table 1:** The characteristics of the participants (*N* = 510)

Variable		n (%)
Gender	Male	33(6.5)
	Female	477(93.5)
Age (M±SD)		34.21±7.65
Educational attainment	Junior College	58 (11.4)
	bachelor’s degree	423 (82.9)
	Master’s degree or above	29 (5.7)
Marital status	Unmarried/Divorced/Widowed	133 (26.1)
	Married	377 (73.9)
Years of work experience	≤2	37(7.3)
	2–5	62(12.2)
	5–10	107(21.0)
	≥10	304(59.6)
Nursing professional ranks	Nurse	57(11.2)
	Senior nurse	149(29.2)
	Supervisor nurse	255(50.0)
	Associate chief nurse or above	49(9.6)
Work Department	Internal Medicine	213(41.8)
	Surgical	153(30.0)
	Critical Care Medicine	144(28.2)
Hospital Classification	Secondary B Hospital	61(12.0)
	Secondary A Hospital	69(13.5)
	Tertiary B Hospital	56(11.0)
	Tertiary A Hospital	324(63.5)
Registered Dietitian Certificate/Health Manager Certificate obtained	No	429(84.1)
	Yes	81(15.9)
Nutrition Specialist Nurse	No	483(94.7)
	Yes	27(5.3)
Attended training in (geriatric) nutritional care	No	400(78.4)
	Yes	110(21.6)
Is it necessary to hold more training sessions on (geriatric) nutritional care	No	64(12.5)
	Yes	446(87.5)
Whether current (geriatric) nutritional care knowledge is sufficient to meet clinical work needs	Not at all sufficient	27(5.3)
	Slightly insufficient	213(41.8)
	Basically sufficient	218(42.7)
	Largely sufficient	39(7.6)
	Completely sufficient	14(2.5)
The primary source of learning about (geriatric) nutrition-related knowledge	Department lectures	130(25.5)
	Academic conferences/lectures	155(30.4)
	Books/brochures	86(16.9)
	Internet	103(20.2)
	Communication with others	36(7.0)
Factors influencing the effectiveness of your (geriatric) nutritional care practice	Insufficient knowledge and lack of standardized management training	333(65.3)
	Irregular or inconsistent doctor’s orders	21(4.1)
	Inconsistent assessments of patients’ nutritional status between doctors and nurses	101(19.8)
	Poor patient compliance	55(10.8)

**Table 2:** Descriptive statistics of SE-NNC scores for participants

Items/Dimensions	M	SD
**Nutritional Care knowledge (NCK)**		
NCK1.Retrieve and understand the available recommendations regarding the management of nutrition alterations among older adults (e.g., guidelines)	3.39	0.988
NCK2.Understand what are the early signs and symptoms of nutrition alterations of among older adults	3.34	0.98
NCK3.Understand how evaluate the basal metabolic rate of older adults	3.34	0.98
NCK4.Understand how correctly determine the daily energy needs of older people according to body weight, physical activity, and clinical conditions	3.31	0.99
NCK5.Understand how to interpret the laboratory tests referred to nutrition alterations (example: albumin, protein C/albumin ratio)	3.30	0.97
NCK6.Understand how to interpret the anthropometric measures and muscle tropism indices	3.26	0.99
Total NCK	19.95	5.37
**Assessment and Evidence Utilization (AEU)**		
AEU7.Evaluate strategies to encourage the consumption of the meal	3.38	0.95
AEU8.Evaluate the strategies for organizing mealtimes in order to promote higher consumption (eg setting, dishes, use of the dining room, instead of feeding the person in the bed)	3.41	0.96
AEU9.Use the available recommendations regarding the management of alterations in the nutritional status for older adults	3.29	0.96
AEU10. Evaluate the nutritional preferences of older patients	3.32	0.95
AEU11.Assess at-risk older for malnutrition	3.40	0.93
AEU12.Identify and evaluate the eating habits of older people	3.35	0.96
AEU13.Detect early signs and symptoms of altered nutritional status in older people	3.29	0.97
AEU14. Identify and evaluate modifiable (e.g. decrease in meal consumption, weight loss, loss of strength) and non-modifiable [e.g. chronic clinical condition, very advanced age ( > 85 years), cognitive and/or disability motor] risk factors of alteration in the nutritional status of older people	3.31	0.96
AEU15.Identify and evaluate the understanding, knowledge and lifestyles (example: nutrition, physical activity, smoking, sedentary lifestyle) of older people	3.35	0.94
AEU16. Evaluate and monitor over time the factors that can influence clinical nutritional outcomes of older people (e.g. frequent or constant failure to take complete meals, loss of strength, weight loss)	3.30	0.97
AEU17.Evaluate and monitor over time the signs and symptoms of impaired nutritional intake of older people	3.31	0.95
AEU18. Evaluate and monitor over time the adherence to recommendations regarding nutrition habits of older people	3.30	0.99
AEU19.Identify and evaluate the appropriate setting to facilitate meal intakes	3.36	0.94
AEU20.Evaluate the nutritional status using validated tools (eg Mini Nutritional Assesement) or alternative methods to estimate the anthropometric measures of older adults (eg mid-arm circumference)	3.32	0.95
AEU21.Ensure the intake of the caloric and protein needs of older people, considering personal and social factors, and clinical conditions	3.26	0.96
Total AEU	49.93	13.24
**Care Delivery (CD)**		
CD22.Educate older persons to manage their clinical condition which can influence nutritional outcomes (example: diabetes mellitus, nephropathy, chronic heart disease)	3.37	0.94
CD23.Support the older person with personalized interventions during the meal if necessary	3.33	0.96
CD24. Ensure the delivery of meals based on the preferences and personal needs of older people	3.33	0.98
CD25. Provide adequate meals both in terms of quality and quantity	3.37	0.95
CD26. Support adequate caloric intake in elderly people who are unable to eat independently (example: establishing the degree of autonomy in feeding, helping people to open food packages, accompanying the person to the table or in the dining room, evaluating the ability to swallow, educating healthcare assistants, volunteers and relatives to recognize signs and symptoms of inhalation)	3.39	0.95
CD27.Organize mealtimes (example: setting and presentation of the food) for facilitating a complete intake	3.45	0.95
Total CD	20.24	5.38
Total	90.12	23.38

### Latent profiles of SE-NNC

**Fig. 1:**
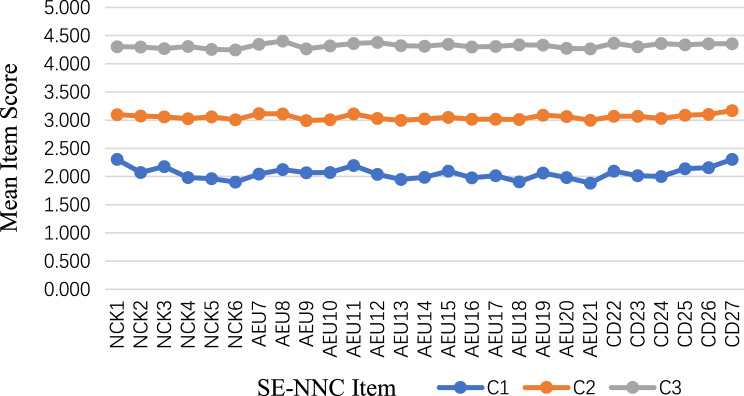
Best-fitting three-class model based on SE-NNC

Table 3 presents the results of the latent profile model fit indices. Although the five-class model showed smaller AIC, BIC, and aBIC values, the BLRT and LMR-LRT tests indicated that both the three-class and four-class models were statistically superior to models with fewer classes. However, the four-class model contained one class with a relatively small sample size (12.5%), limiting interpretability. Considering model fit, parsimony, and practical applicability, the three-class model was selected as the best-fitting solution. This model showed the highest entropy (0.992) and a class distribution that allowed meaningful interpretation (15.9%, 49.2%, 34.9%). The distribution and conditional means of SE-NNC items for each class are presented in Table 4 and Fig. 1. The three classes were defined as C1 “low SE-NNC” (*n* = 81, 15.9%), C2 “moderate SE-NNC” (*n* = 251, 49.2%), and C3 “high SE-NNC” (*n* = 178, 34.9%).

**Table 3:** Model fit indices for LPA model

Clsass	k	Log(L)	AIC	BIC	aBIC	Entropy	*p***LMR**	*p***BLRT**	Profile Probability (%)
1	54	−18971.014	38050.028	38278.687	38107.283	-	-	-	-
2	82	−13526.284	27216.568	27563.789	27303.510	0.897	< 0.001	< 0.001	0.633/0.367
3	110	−11257.689	22735.378	23201.163	22852.008	0.992	< 0.001	< 0.001	0.159/0.492/ 0.349
4	138	−9375.197	19026.393	19610.742	19172.710	0.990	0.024	< 0.001	0.153/0.475/ 0.125/0.247
5	166	−8963.824	18259.648	18962.560	18435.653	0.995	0.239	< 0.001	0.016/0.155/ 0.465/0.239/ 0.125

**Table 4:** Conditional means of items of SE-NNC on each class

Item	C1	C2	C3
NCK1	2.303	3.097	4.303
NCK2	2.070	3.073	4.298
NCK3	2.178	3.058	4.270
NCK4	1.984	3.025	4.309
NCK5	1.962	3.059	4.254
NCK6	1.901	3.009	4.246
AEU7	2.044	3.117	4.348
AEU8	2.123	3.113	4.403
AEU9	2.069	2.993	4.264
AEU10	2.072	3.005	4.319
AEU11	2.194	3.113	4.359
AEU12	2.037	3.033	4.381
AEU13	1.948	2.997	4.320
AEU14	1.988	3.021	4.313
AEU15	2.095	3.050	4.347
AEU16	1.978	3.019	4.298
AEU17	2.015	3.016	4.308
AEU18	1.908	3.014	4.337
AEU19	2.064	3.087	4.331
AEU20	1.984	3.066	4.274
AEU21	1.884	2.997	4.264
CD22	2.094	3.069	4.365
CD23	2.014	3.067	4.303
CD24	2.001	3.032	4.359
CD25	2.138	3.087	4.337
CD26	2.158	3.104	4.354
CD27	2.304	3.170	4.353

### Four Ising networks

**Fig. 2:**
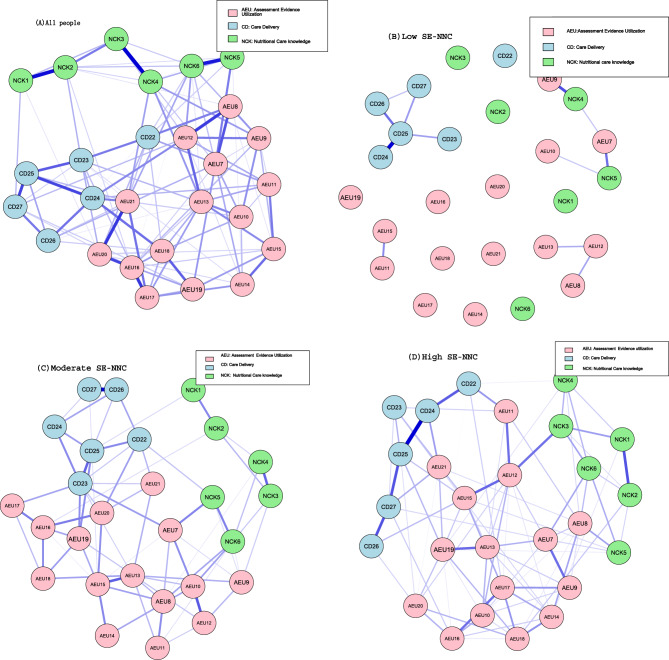
Ising networks estimated from responses of the full study population. (**A**) all people; (**B**) low SE-NNC; (**C**) moderate SE-NNC; (**D**) high SE-NNC. Note: node characteristics are shown in Table 2

Figure 2 shows the SE-NNC network structures for the overall sample and the three SE-NNC classes (low, moderate, high) identified by LPA. Nodes represent assessment and evidence utilization (pink), care delivery (light blue), and nutritional care knowledge (light green). Edges represent logistic regression coefficients, with purple edges indicating positive correlations. All connections were positive, and edge thickness reflects coefficient magnitude.

**Fig. 3:**
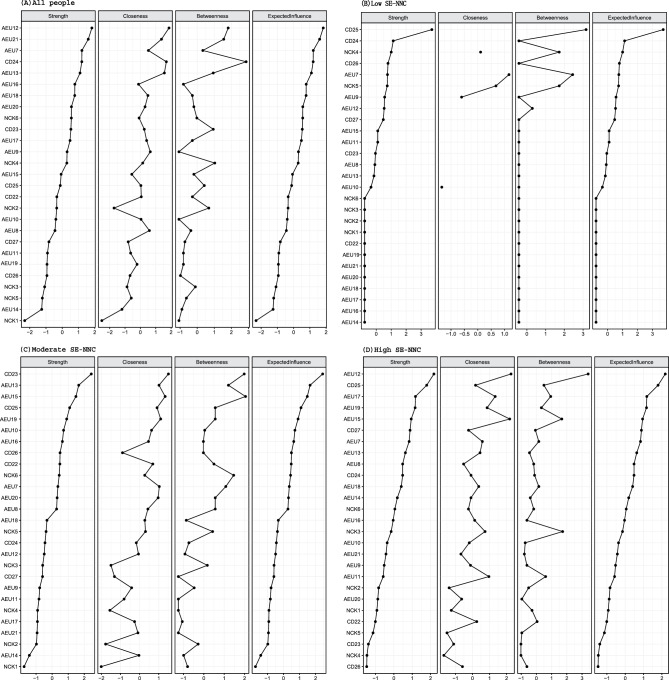
Bridge centrality indices of nodes shown as standardized values z scores. (**A**) all people; (**B**) low SE-NNC; (**C**) moderate SE-NNC; (**D**) high SE-NNC

Centrality indices are shown in Fig. 3. In the overall sample, AEU12 (identify and evaluate the eating habits of older adults) exhibited the highest centrality, with strength and expected influence z-scores of 1.795. In the high SE-NNC, AEU12 also ranked highest (1.854). In the moderate SE-NNC, CD23 (support older adults with personalized interventions during the meal) had the highest centrality (2.787), while in the low SE-NNC, CD25 (provide adequate meals in quality and quantity) was most central (3.675). Bootstrap 95% confidence intervals were relatively narrow for the overall, high, and moderate networks. In the low SE-NNC network, however, the confidence intervals were wider, indicating less precise edge estimates (see Supplementary Figure 1). Results for the low SE-NNC network should thus be interpreted with caution. CS-C for all networks ranged from 0.26 to 0.28, representing the minimum acceptable range of 0.25–0.50 (see Supplementary Figure 2).

To validate the Ising model findings, Gaussian Graph Models (GGM) were fitted using the original continuous SE-NNC scores (Supplementary Figure 3). The top central nodes in the GGM networks differed slightly from the Ising networks. AEU12 remained among the top three in the overall sample and high SE-NNC, indicating consistent importance. In the moderate SE-NNC, the central node shifted to AEU15, while in the low SE-NNC, the central node shifted from CD25 to AEU16, highlighting changes in core nodes across these subgroups. Centrality indices for each network are shown in Supplementary Figure 4, with bootstrap 95% confidence intervals (Supplementary Figure 5) and CS-C values (Supplementary Figure 6) indicating overall stable estimates. The specific GGM results are provided in Supplementary Material 2.

### Effects of computer-simulated interventions

**Fig. 4:**
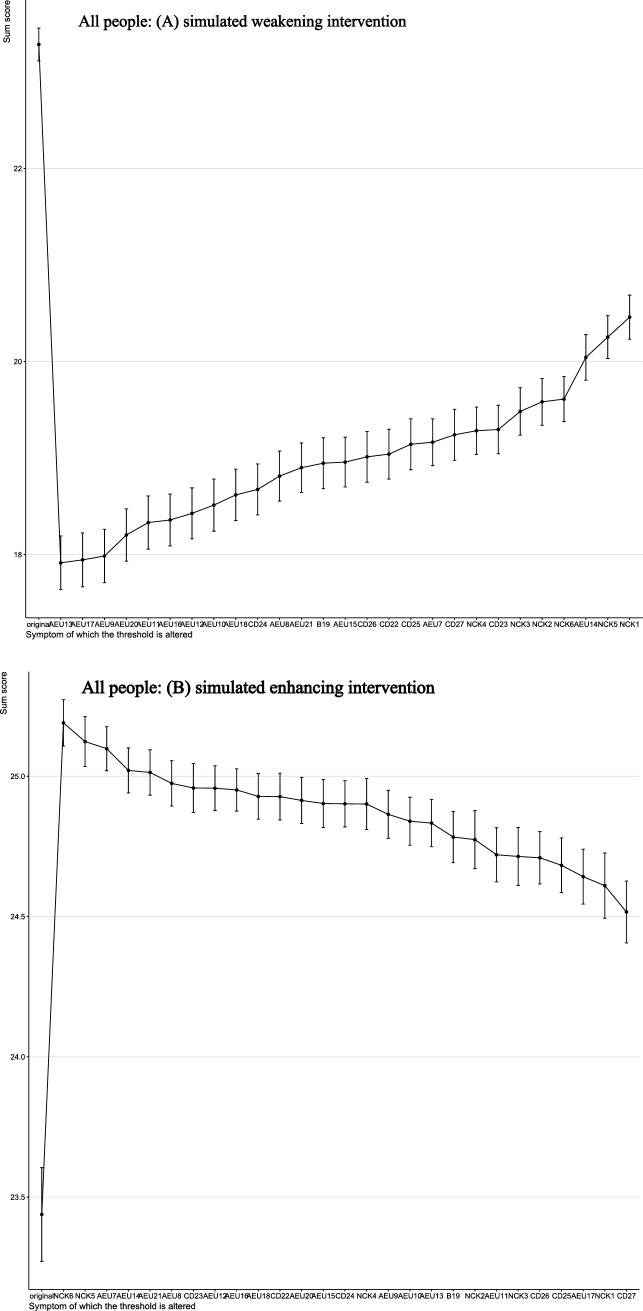
Changes in total SE-NNC scores (based on binary-transformed items) following simulated interventions in all people

**Fig. 5:**
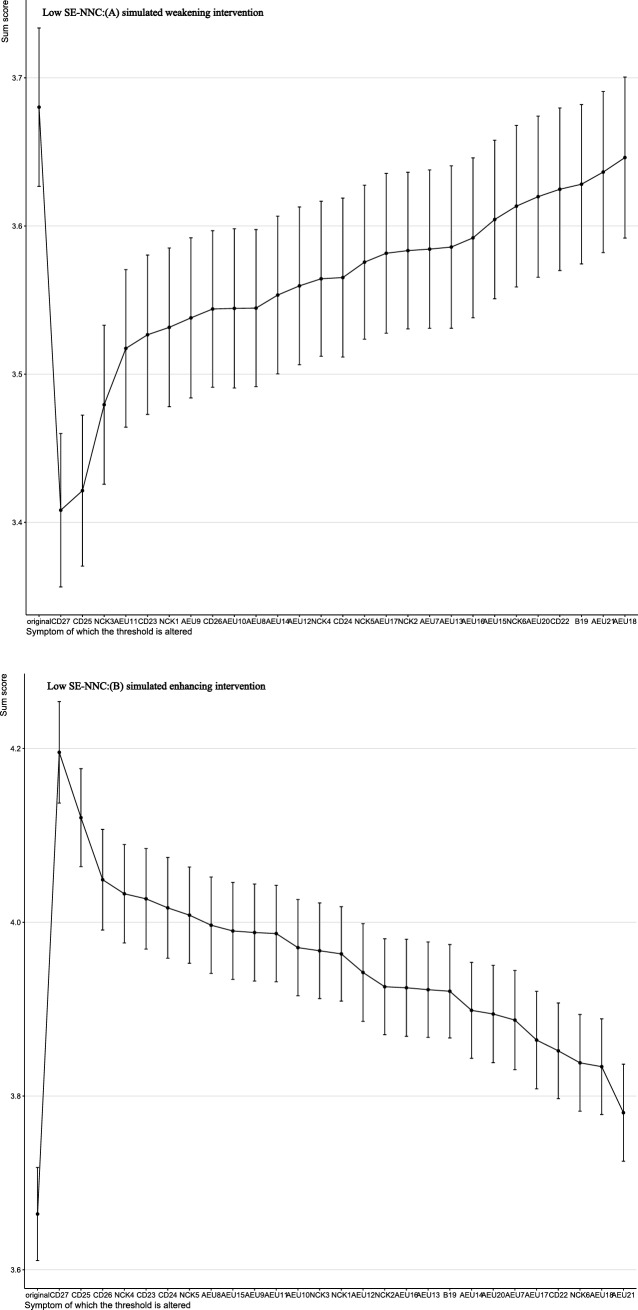
Changes in total SE-NNC scores (based on binary-transformed items) following simulated interventions in the low SE-NNC

**Fig. 6:**
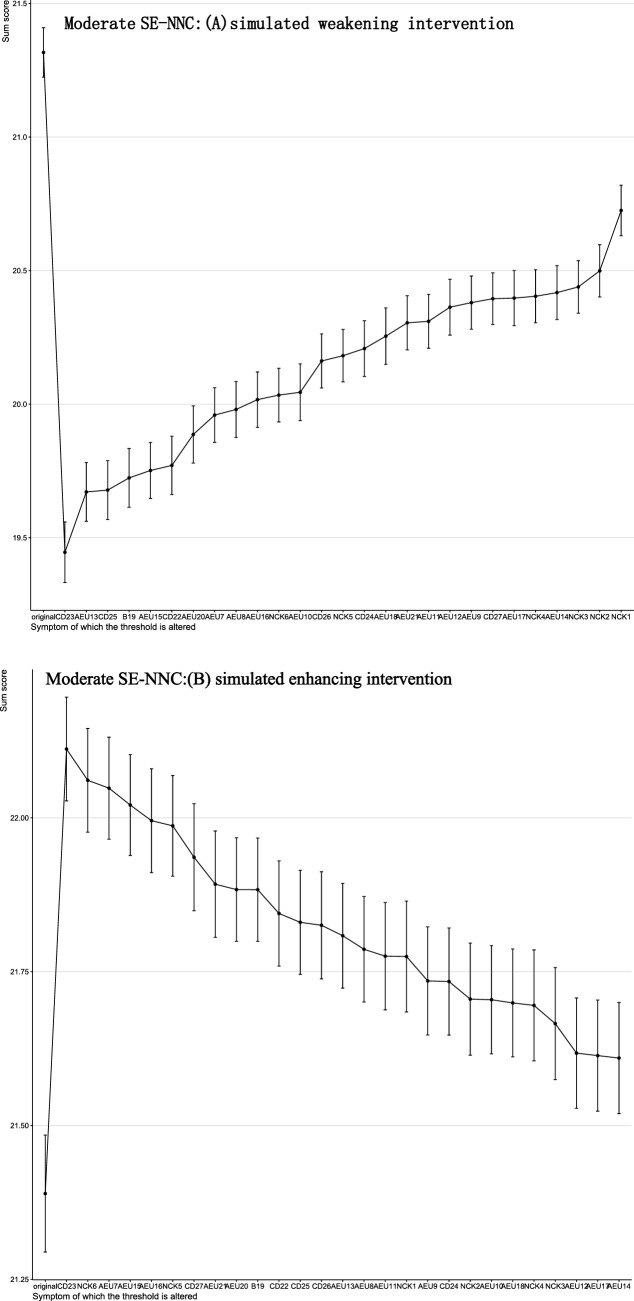
Changes in total SE-NNC scores (based on binary-transformed items) following simulated interventions in moderate SE-NNC

**Fig. 7:**
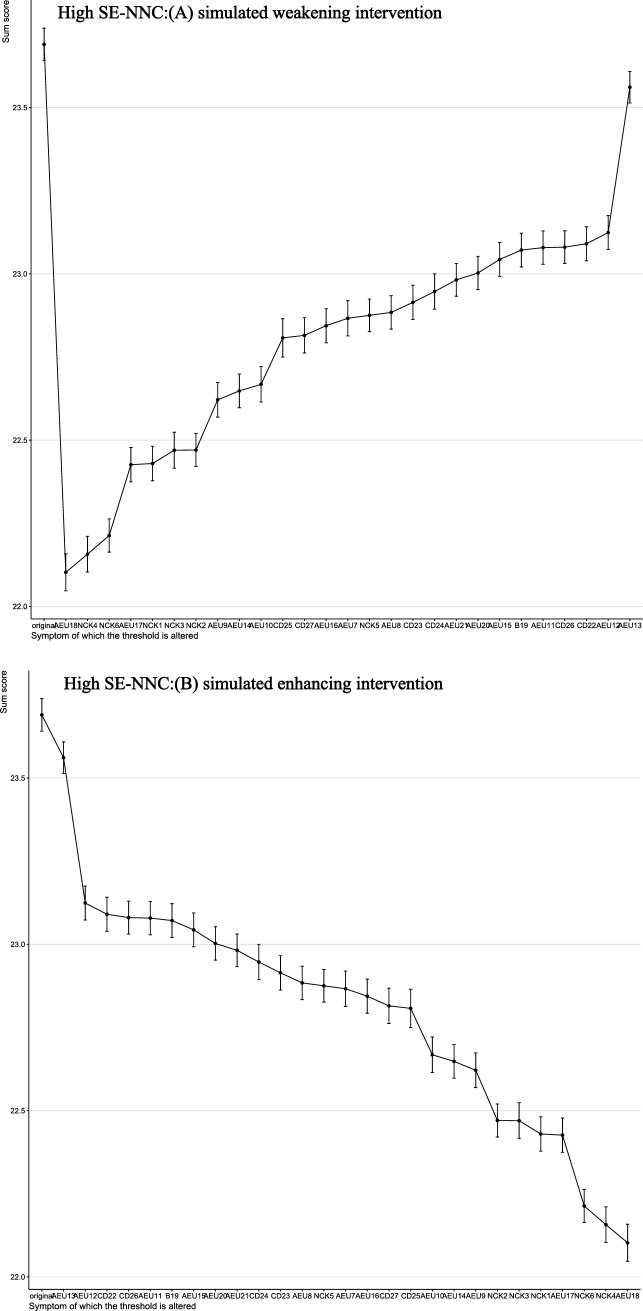
Changes in total SE-NNC scores (based on binary-transformed items) following simulated interventions in high SE-NNC

The results of the computer-simulated interventions are summarized in Figs. 4–7. In these analyses, the total score represents a network-derived summary metric based on binary-transformed SE-NNC items, rather than the raw SE-NNC score. At baseline, the network-derived total score was 23.28 under simulated weakening and 23.44 under simulated enhancing in the overall population. In low SE-NNC, the baseline scores were 3.68 and 3.66, respectively; in moderate SE-NNC, 21.32 and 21.39; and in high SE-NNC, 23.69. In the overall sample (Fig. 4), simulated weakening and enhancing interventions identified distinct key targets. Targeting AEU13 (detect early signs and symptoms of altered nutritional status in older people) produced the greatest reduction in the network-derived total score, whereas targeting NCK6 (interpret anthropometric measures and muscle tropism indices) resulted in the largest increase.

In participants with low SE-NNC (Fig. 5), CD27 (organize mealtimes to facilitate complete intake) appeared to have a relatively large effect under both simulated weakening and enhancing interventions. In moderate SE-NNC (Fig. 6), CD23 (support the older person with personalized interventions during meals when necessary) was associated with comparatively larger simulated changes. In high SE-NNC (Fig. 7), AEU18 (monitor adherence to nutritional recommendations over time) and AEU13 were identified as nodes contributing notably to the simulated outcomes. Overall, the intervention targets differed according to SE-NNC status, suggesting that different nodes may play a more prominent role depending on the underlying SE-NNC score. Specific numerical changes in the network-derived total scores are provided in Supplementary Material 3.

## Discussion

This study is the first to characterize the network structure of nurses’ self-efficacy in providing nutritional care for older adults. It integrates LPA with network analysis and further examines the LPA-based networks through computer-simulated interventions.

LPA identified three classes: low, moderate, and high, indicating the presence of heterogeneous SE-NNC experiences. Although the core traits identified by the Ising model (based on binarized data) differed slightly from those in the GGM (based on continuous data), the differences were minimal. In the total population as well as in moderate and high SE-NNC, Ising model features consistently ranked among the top three in both models. This pattern indicates robust and consistent results, which are similar to findings reported in a previous study [25].

Greater variation was observed in low SE-NNC, where the core node shifted from CD25 (provide adequate meals in terms of quality and quantity) in the Ising model to AEU16 (evaluate and monitor over time the factors influencing older adults’ clinical nutritional outcomes) in the GGM network. This shift may partly result from reduced network stability due to the smaller sample size [17]. Additionally, CD25 belongs to the care delivery dimension, whereas AEU16 falls under assessment and evidence utilization. The transition of the core node may indicate that nurses with lower self-efficacy may focus more on recognizing and assessing nutritional risks rather than implementing direct interventions. Clinically, nutritional assessment provides the foundation for identifying high-risk or malnourished older adults. Confidence in these evaluative skills is essential for effective intervention [2, 10]. Therefore, strengthening nurses’ training in nutrition assessment, screening, and continuous monitoring could be a potential strategy to enhance both nutritional care competence and self-efficacy of this class.

Notably, the computer-simulated interventions analysis provides important theoretical insights into potential key nodes for enhancing or weakening SE-NNC. However, these findings do not constitute causal evidence and should therefore be interpreted with caution [17]. In the total population, AEU13 (detect early signs and symptoms of altered nutritional status in older people) was identified as a potential preventive target for weakening nurses’ nutrition care self-efficacy. One possible explanation is that nurses often lack awareness of using effective nutritional assessment tools, such as the Mini-Nutritional Assessment (MNA) or the Malnutrition Universal Screening Tool (MUST). This lack of awareness can lead to failure to timely identify nutritional risks in older adults [14, 26, 27]. Therefore, implementing on-the-job training programs led by senior nurses is recommended. These programs could include systematic nutrition screening and assessment methods, instruction on relevant guidelines, and the establishment of structured observation or monitoring checklists [14].

In addition, NCK6 (understand how to interpret anthropometric measures and muscle tropism indices) was identified as a potential intervention target for enhancing self-efficacy, within the domain of nutrition knowledge. Insufficient nutritional knowledge is widely recognized as a key barrier to high-quality nutrition practices. Nurses lacking this knowledge often struggle to develop and implement effective nutrition care plans [7, 14]. To address this issue, three recommendations are proposed to improve both nurses’ knowledge and practical competence. First, develop and promote high-quality, evidence-based training programs on malnutrition, such as Massive Open Online Courses (MOOCs) for healthcare professionals [28, 29]. Second, encourage the implementation of cross-national, evidence-informed nutrition care guidelines to facilitate translation of knowledge into practice [7]. Third, consider feasibility constraints in implementation, including limitations in equipment, procedural training, or testing capacity, as well as high nurse workload. When necessary, employ practical alternatives, such as brief nutrition screening tools, handgrip strength assessment, or monitoring weight changes [2].

In both low and moderate SE-NNC, CD27 (organize mealtimes for facilitating a complete intake) and CD23 (support the older person with personalized interventions during meals if necessary) consistently demonstrated relatively large effects under both simulated weakening and enhancing conditions. Such consistency of key nodes across different simulation scenarios is uncommon in previous studies [17, 30], suggesting that these intervention targets may be robust and potentially valuable.

The behavior of organizing regular mealtimes involves planning, communication, and understanding the importance of nutrition [31]. This represents an area where individuals with low SE-NNC may show weaknesses. It is recommended to pay special attention to this class and enhance their comprehensive abilities in mealtime planning, patient communication, and application of nutritional knowledge. This can be achieved through scenario-based training, interdisciplinary collaborative exercises, and case-based reflective learning [32], which may improve their self-efficacy in nutrition care. Supporting older adults with personalized care during mealtimes is a key intervention target for moderate SE-NNC. Implementing personalized care plays a significant role in preventing malnutrition in older adults. However, in practice, high workloads and the structural setup of hospital wards often limit nurses’ ability to provide individualized care [14, 33]. It is recommended to strengthen interdisciplinary collaboration, improve the working environment, and adjust workflows appropriately to optimize their working conditions [14, 31].

In the high SE-NNC, AEU18 (evaluate and monitor over time the adherence to recommendations regarding nutrition habits of older people) and AEU13 were identified as nodes with relatively large effects for weakening and enhancing SE-NNC, respectively. Notably, AEU13 acted as an enhancing target in this class but appeared as a weakening factor in the overall sample. This highlights that intervention priorities may differ across groups with varying levels of self-efficacy. AEU18 was identified as a node potentially important for weakening SE-NNC, suggesting that even nurses with high self-efficacy may face challenges in evaluating and monitoring older adults’ adherence to nutritional recommendations over time. Continuous monitoring often requires systematic procedures, interdisciplinary collaboration, and time-consuming follow-up, which may pose practical limitations in clinical settings [34]. It is recommended to explore ways to improve nurses’ efficiency and access to resources for monitoring nutritional adherence, for example, by incorporating artificial intelligence tools [35].

Overall, the study suggests several strategies to enhance nurses’ self-efficacy in nutritional care. First, systematic training on nutritional assessment tools, such as the MNA or the MUST, combined with structured observation or monitoring checklists, may strengthen nurses’ ability to identify nutritional risks in older adults. Second, developing evidence-based nutrition training programs, promoting interdisciplinary collaboration, and considering practical feasibility could help nurses design effective nutritional care plans and improve professional judgment. Finally, improving working conditions, introducing artificial intelligence technologies, and fostering teamwork may facilitate the implementation of personalized mealtime interventions. These measures could also help nurses organize regular meals more effectively, thereby potentially reinforcing their confidence and competence in nutritional care.

It should be noted that several hospital-level factors need to be considered when interpreting the findings of this study, including case mix, average length of stay, staffing, and availability of nutrition services. First, most nurses were highly educated (88.6% with a bachelor’s degree or higher) and had substantial clinical experience (59.6% with ≥10 years of work). However, the participation rate in geriatric nutrition training was relatively low (78.4%), even though most perceived a need for further training (87.5%). This gap between general nursing competence and specific nutrition care experience may influence the latent profile structure. It may also lead to greater variability in node weights within the network analysis for the low SE-NNC. Second, 63.5% of participants were employed in tertiary A hospitals. These hospitals primarily treat acute and critically ill patients, with complex case mixes, shorter lengths of stay, and limited staffing. As a result, nurses have relatively fewer opportunities to provide comprehensive, personalized nutritional care [11].

This study has several limitations. First, in the low SE-NNC, network node weights fluctuated considerably due to the small sample size, resulting in relatively lower stability of centrality and edge weights. Future studies should increase sample sizes for specific groups and explore more precise modeling methods to enhance the robustness and reliability of network estimates. Second, although this study provides valuable theoretical insights, its practical applicability in clinical settings and causal relationships remain to be validated. Longitudinal or interventional studies are needed to assess the real-world relevance of these findings. Third, the survey was conducted only in Jiangsu Province, an economically developed region with abundant medical resources. Therefore, the findings may not generalize to nurses in less developed regions. Future studies should include nurses from diverse regions to evaluate the broader applicability of the results. Finally, differences across nurse demographic characteristics (e.g., professional title, department) were not examined. Future research could investigate how these factors influence classification and SE-NNC development, providing a more comprehensive understanding of self-efficacy variations among nurses with different backgrounds.

## Conclusions

This study used LPA to classify SE-NNC into three classes: low, moderate, and high. Two simulated intervention models identified five key nodes: NCK6 (understand how to interpret the anthropometric measures and muscle tropism indices), AEU13 (detect early signs and symptoms of altered nutritional status in older people), AEU18 (evaluate and monitor over time the adherence to recommendations regarding nutrition habits of older people), CD23 (support the older person with personalized interventions during the meal if necessary), and CD27 (organize mealtimes, e.g., setting and presentation of the food, for facilitating a complete intake). These nodes exhibited distinct response patterns across different self-efficacy classes, highlighting the need for stratified, targeted interventions in practice. The findings suggest that incorporating self-efficacy–based strategies into continuing and in-service nursing education can enhance nurses’ core competencies in geriatric nutrition and better prepare them to address emerging clinical challenges.

## Electronic supplementary material

Below is the link to the electronic supplementary material.

Supplementary Material 1

## Data Availability

Materials and analysis code for this study are available by emailing the corresponding author upon reasonable request.
