# Supplementary material for: Self-efficacy in nutritional care for older adults among hospital-based nurses: a latent profile and network simulation analysis
Source: BMC Nurs. 2026 Feb 12;25:245. doi: 10.1186/s12912-026-04391-8 (PMC12998110; doi:10.1186/s12912-026-04391-8)
Supplement: Supplementary file 1 — Supplementary Material 1 [file 12912_2026_4391_MOESM1_ESM.docx]

**Supplementary materials**

**Supplementary Material 1** Data Analysis

All data processing in the network analysis was performed in R 4.2.1 within RStudio, using the R packages "haven", "bootnet", "networktools", "nodeIdentifyR", and "dplyr". Since there were no missing values in the data, no missing value handling was performed.

Each item in the SE-NNC was conceptualized as a node. This study constructed an SE-NNC network consisting of 27 nodes. Through latent profile analysis, the SE-NNC was divided into three classes: low SE-NNC, moderate SE-NNC, and high SE-NNC. Therefore, a total of four SC-NNE networks were constructed, including one overall network for all people and three networks corresponding to each class.

Because computer-simulated interventions function by adjusting the intercept term—also known as the threshold parameter—in the logistic regression model of a specific node relative to all other nodes, fitting the Ising model is required for this process (Lunansky et al., 2022). The Ising model requires binary data. However, the SE-NNC uses a 5-point Likert scoring system, where 1, 2, 3, 4, and 5 correspond to " not confidence at all," "Slightly confident," " Moderately confident," " Very confident," and "Completely confident," respectively. Therefore, items reported as " not confidence at all " was coded as 0, indicating the absence of the corresponding SE-NNC trait; items reported as "Slightly confident," " Moderately confident," " Very confident," and "Completely confident," were coded as 1, indicating the presence of the SE-NNC trait.

The estimateNetwork function from the bootnet package was first used to fit the Ising model (SE-NNC network) for the total population and the three classes. To explore which features in the four SE-NNC networks are core traits, four centrality indices were calculated: strength, expected influence, closeness, and betweenness [1]. However, previous research suggested that closeness and betweenness are less sensitive in psychometrics [1]. Therefore, this study focused solely on strength and expected influence, where higher values indicated stronger connections with other nodes, reflecting greater importance in the network and identifying core traits.

To assess the stability of the centrality indices, a stability test was further conducted, primarily measured by the central stability coefficient (CS-C). A CS-C greater than 0.5 indicates strong stability, a CS-C between 0.25 and 0.50 indicates acceptable stability, and a CS-C less than 0.25 indicates poor stability of the measure [1]. In addition, considering that the Ising network model may lose some of the original data information, a Gaussian graph model was also constructed, and the centrality indices were calculated, while the stability of these indices was checked to validate the results obtained from the Ising model.

Next, computer-simulated interventions were performed using the nodeIdentifyR algorithm (NIRA), which offers two types of interventions: alleviating interventions and aggravating interventions [2-4]. According to previous studies, alleviating interventions reduce the trait threshold by subtracting two standard deviations from the original threshold parameter, whereas aggravating interventions increase the trait threshold by adding two standard deviations to the original parameter [2-4]. In this study, we considered it more appropriate to refer to these two types of interventions as “enhancing interventions” and “weakening interventions,” respectively.

Then, two types of simulated interventions (enhancing and weakening interventions) were applied to each node in the SE-NNC network of the total population.The total SE-NNC values in the samples after the simulated interventions were calculated, and the changes in SE-NNC levels in the samples across 28 simulations (1 original simulation + 27 simulated interventions) were plotted.

Finally, we repeated the above steps sequentially in the three classes of SE-NNC.

Reference

[1] Epskamp S, Borsboom D, Fried EI. Estimating psychological networks and their accuracy: A tutorial paper. Behav Res Methods. 2018;50(1):195-212. doi: 10.3758/s13428-017-0862-1.

[2] Lunansky G, Naberman J, van Borkulo CD, Chen C, Wang L, Borsboom D. Intervening on psychopathology networks: Evaluating intervention targets through simulations. Methods. 2022;204:29-37. doi: 10.1016/j.ymeth.2021.11.006.

[3] Machado GM, Skjeldal KE, Grønnerød C, de Carvalho LF. Comparing NIRA and Traditional Network Approaches: A Study Case With Antisocial Personality Disorder Traits. J Pers. 2024. doi: 10.1111/jopy.13005.

[4] Liang M, Pan Y, Cai J, Xiong Y, Liu Y, Chen L, Xu M, Zhu S, Mei X, Zhong T, Knobf MT, Ye Z. Navigating specific targets of breast cancer symptoms: An innovative computer-simulated intervention analysis. Eur J Oncol Nurs. 2025 ;74:102708. doi: 10.1016/j.ejon.2024.102708.


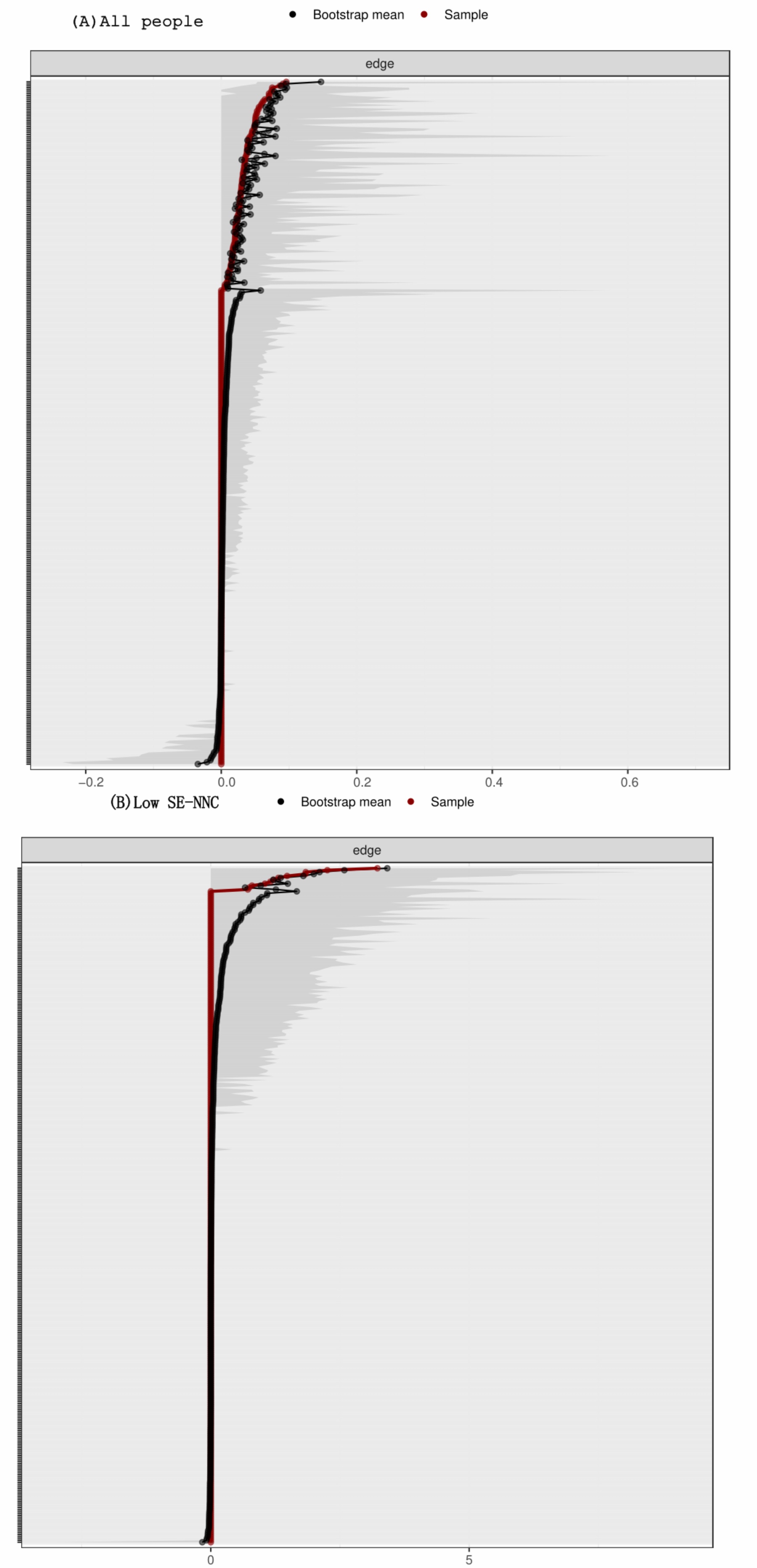


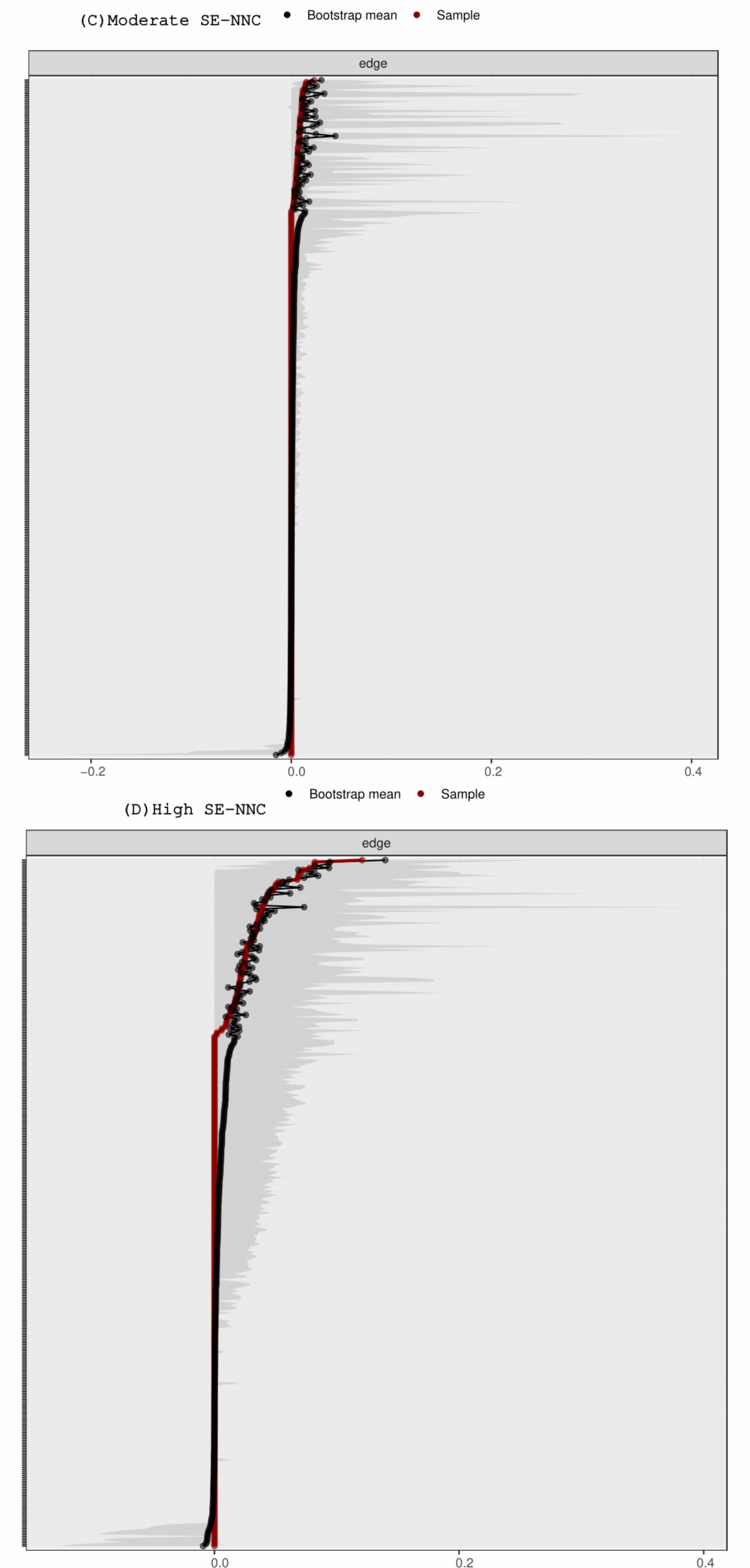


**Supplementary Figure 1** The 95% CIs of the edge weights in Ising network: (A) All people; (B) Low SE-NNC; (C) Moderate SE-NNC; (D) High SE-NNC.


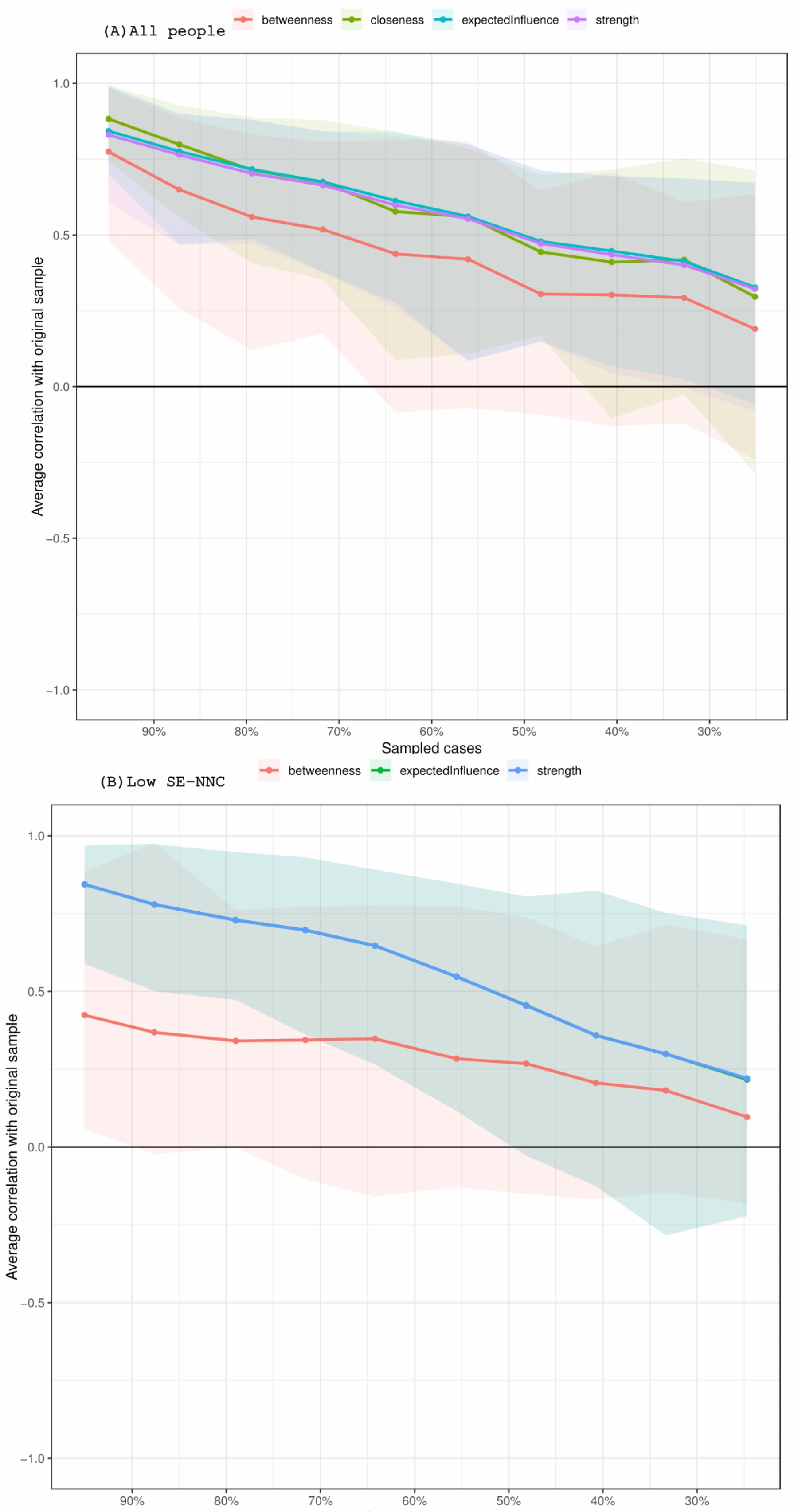


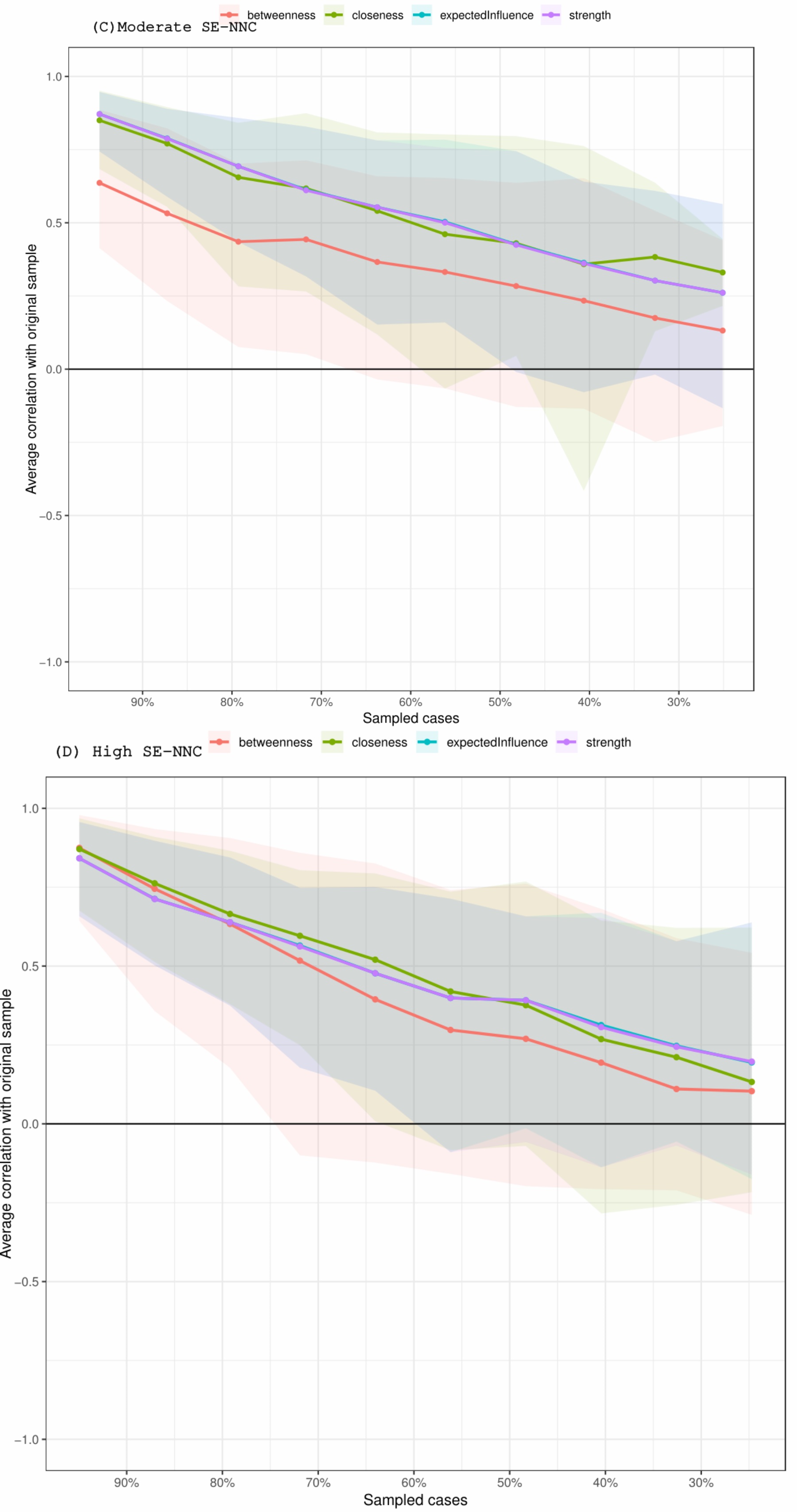


**Supplementary Figure 2** Stability test of 1000 bootstrap tests for centrality index in Ising Network : (A) All people; (B) Low SE-NNC; (C) Moderate SE-NNC; (D) High SE-NNC.


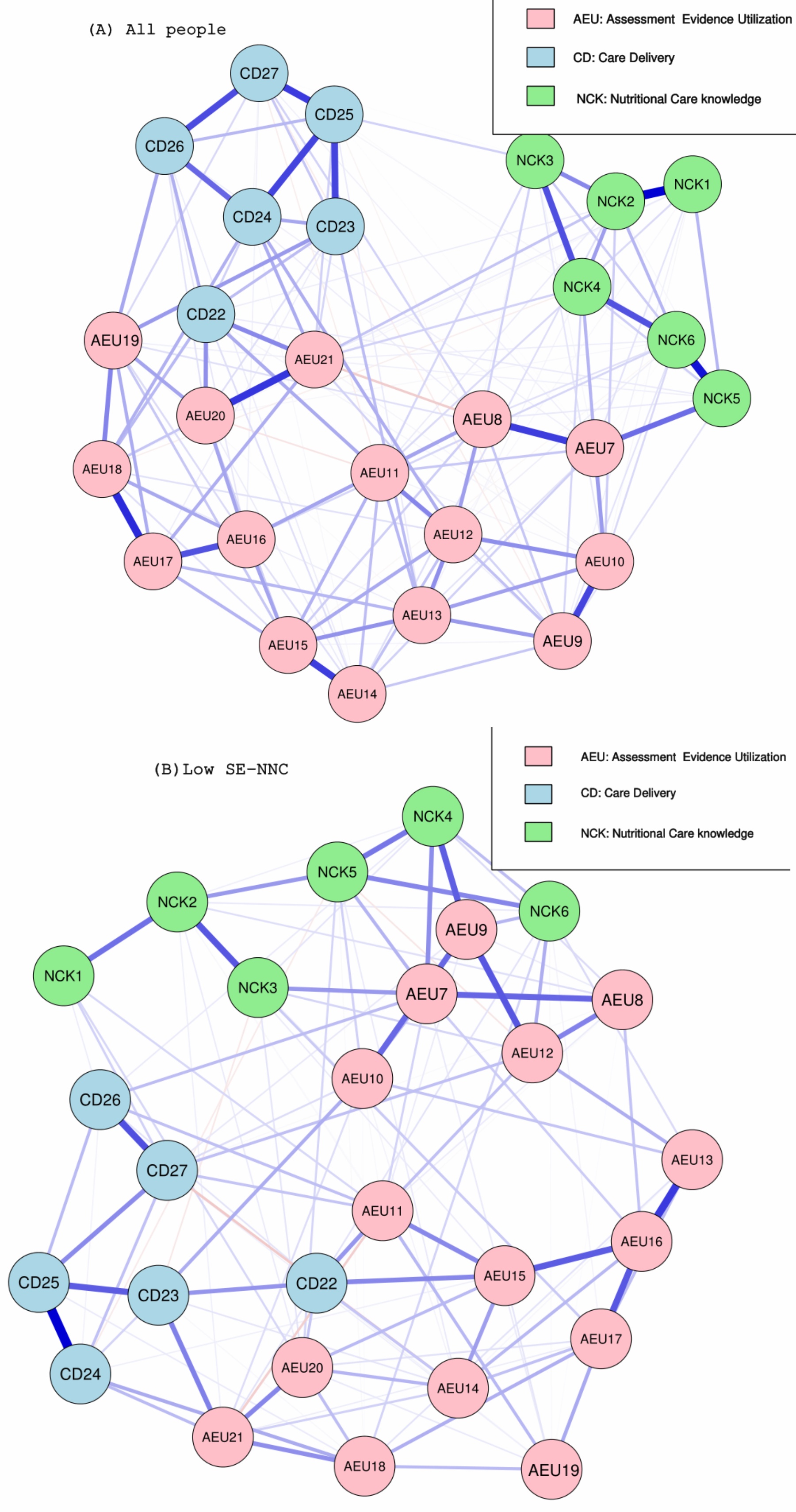


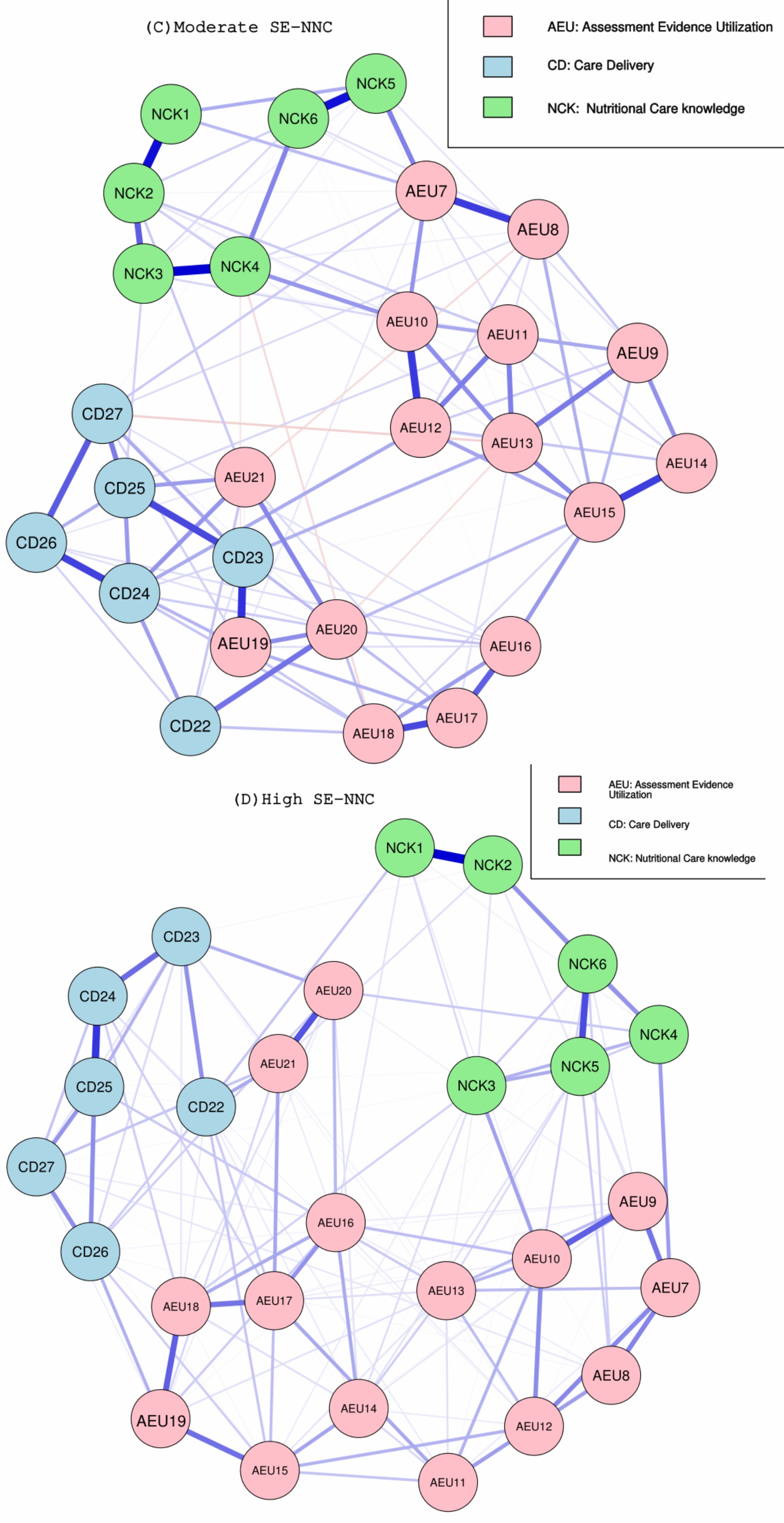


**Supplementary Figure 3** GGM networks estimated from responses of the study population: (A) All people; (B) Low SE-NNC; (C) Moderate SE-NNC; (D) High SE-NNC.


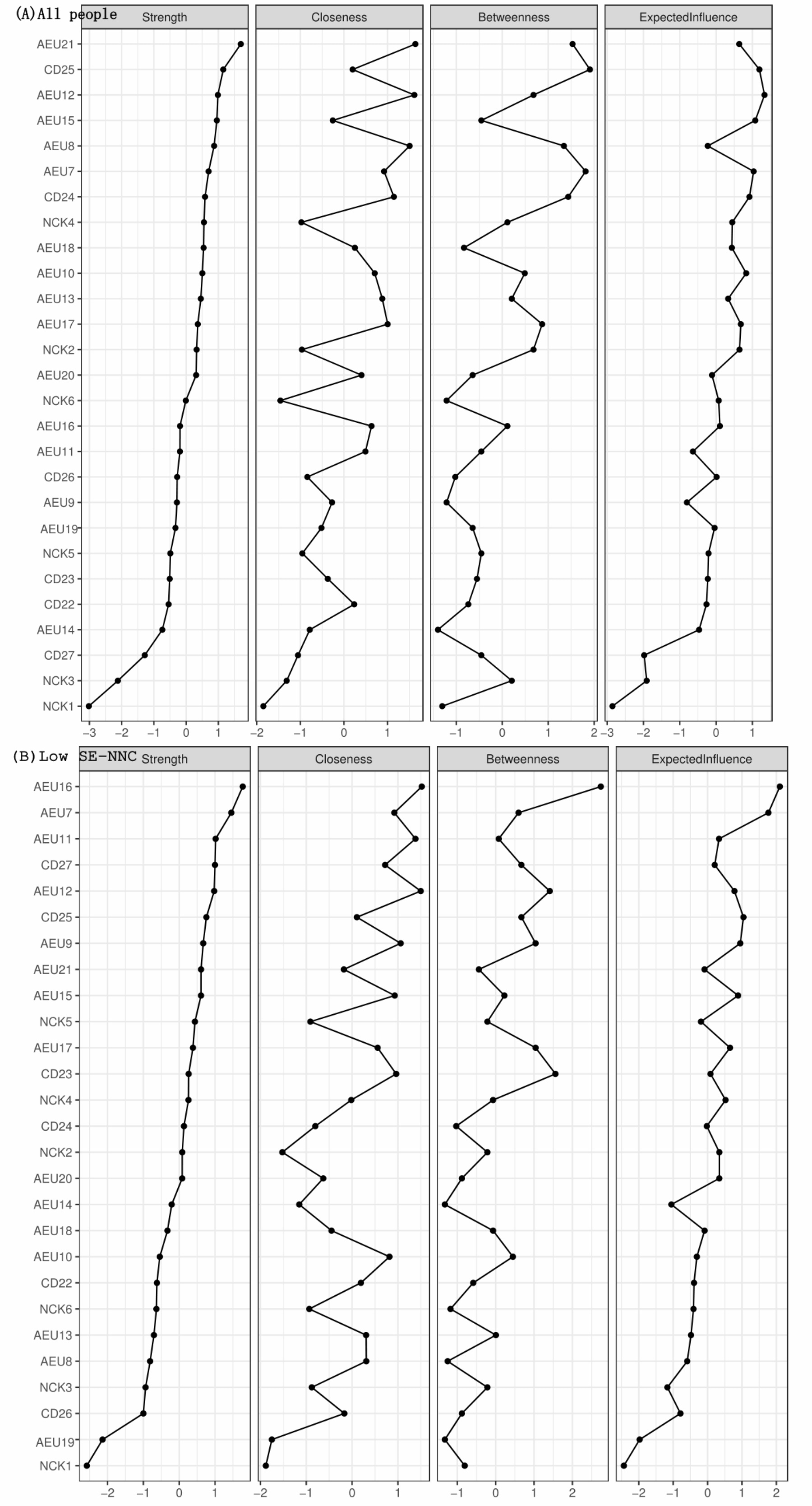


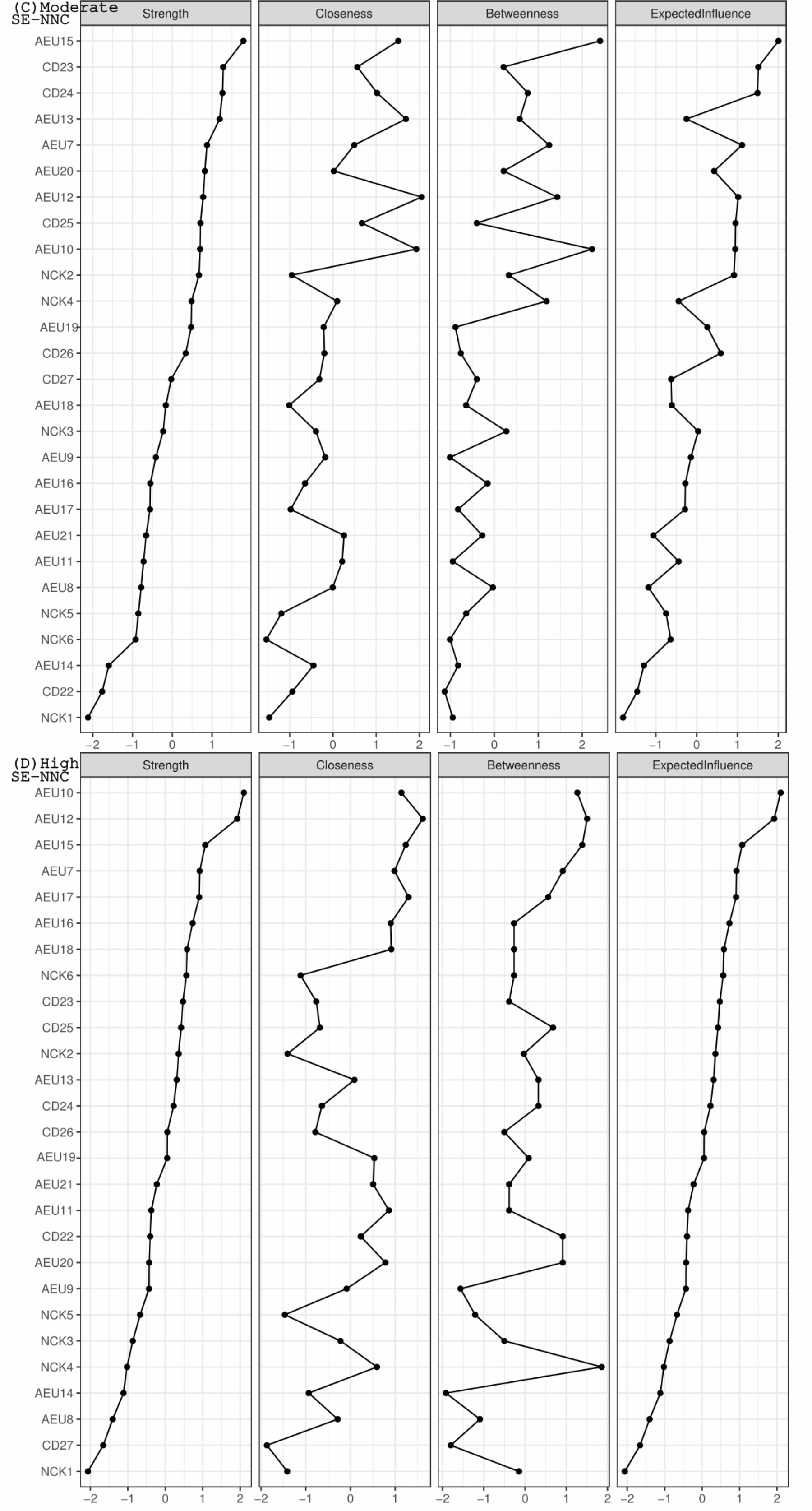
**Supplementary Figure 4** Centrality indices in GGM network: (A) All people; (B) Low SE-NNC; (C) Moderate SE-NNC; (D) High SE-NNC.

**Supplementary Material 2**

In the overall sample, the node with the highest strength in the GGM network was AEU21 (ensure the intake of caloric and protein needs of older people, considering personal, social factors, and clinical conditions), with a strength of 1.197, expected influence of 1.047, and CS-C of 0.361 and 0.594, respectively. In the high SE-NNC, the node with the highest centrality was AEU10 (evaluate the nutritional preferences of older patients), with strength and expected influence of 1.204, and CS-C values of 0.438 for both indices. Although the top-ranking central nodes differed from those in the Ising networks, AEU12 (identify and evaluate the eating habits of older adults)—which was the most central node in the Ising model—remained among the top three in the GGM networks, highlighting its consistent importance across modeling approaches.

In the low SE-NNC, the central node shifted from CD25 to AEU16 (evaluate and monitor over time the factors that can influence clinical nutritional outcomes of older adults), with both strength and expected influence equal to 1.251 and CS-C of 0.25. In the moderate SE-NNC AEU15 (identify and evaluate the understanding, knowledge, and lifestyles of older adults) became the most central node, with strength and expected influence both 1.128 and CS-C of 0.283, while CD23—the core node in the Ising network—still ranked second in the GGM network for this class, indicating a certain degree of consistency between the two models.

**Supplementary Material 3** Results: Effects of computer-simulated interventions

The baseline total SE-NNC score was 23.28 (simulated weakening intervention) or 23.44 (simulated enhancing intervention) in overall population. AEU13 (detect early signs and symptoms of altered nutritional status in older people) was the key target in the simulated weakening intervention, reducing the total score to 17.91 (a decrease of 5.37 points, approximately 23.1%), whereas NCK6 (understand how to interpret the anthropometric measures and muscle tropism indices) was the key target in the simulated strengthening intervention, increasing the total score to 25.19 (an increase of approximately 7.4%).

In the low SE-NNC (C1), the baseline total score was 3.68 (simulated weakening) or 3.66 (simulated enhancing). CD27 (organize mealtimes for facilitating a complete intake) was the key target, and under simulated intervention, it could reduce the total score to 3.41 (a decrease of 0.27 points, approximately 7.3%) or increase it to 4.20 (an increase of approximately 14.4%), demonstrating its robust role in regulating low SE-NNC.

In the moderate SE-NNC (C2), the baseline total score was 21.32 (simulated weakening) or 21.39 (simulated enhancing). CD23 (support the older person with personalized interventions during the meal if necessary) was the key target, and under simulated intervention, it could reduce the total score to 19.45 (a decrease of 1.87 points, approximately 8.8%) or increase it to 21.99 (an increase of approximately 2.9%), showing its central role in regulating C2.

In the high SE-NNC (C3), the baseline total score was 23.69. AEU18 (evaluate and monitor over time the adherence to recommendations regarding nutrition habits of older people) and AEU13 were the key targets in the simulated weakening and enhancing interventions, respectively, reducing the total score to 22.10 (a decrease of 1.59 points, approximately 6.7%) or increasing it to 23.56 (an increase of approximately 3.0%), demonstrating their important role in regulating C3.


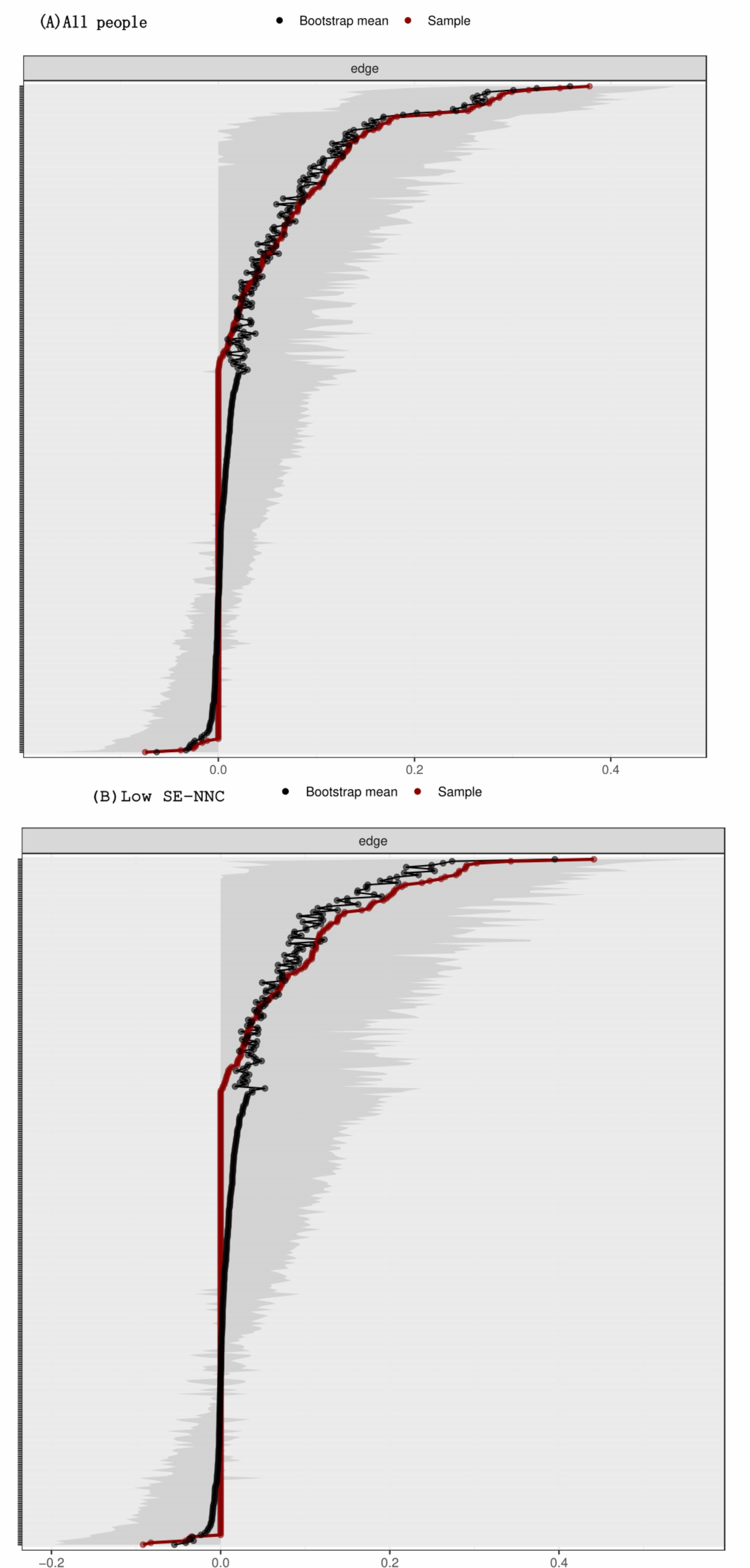


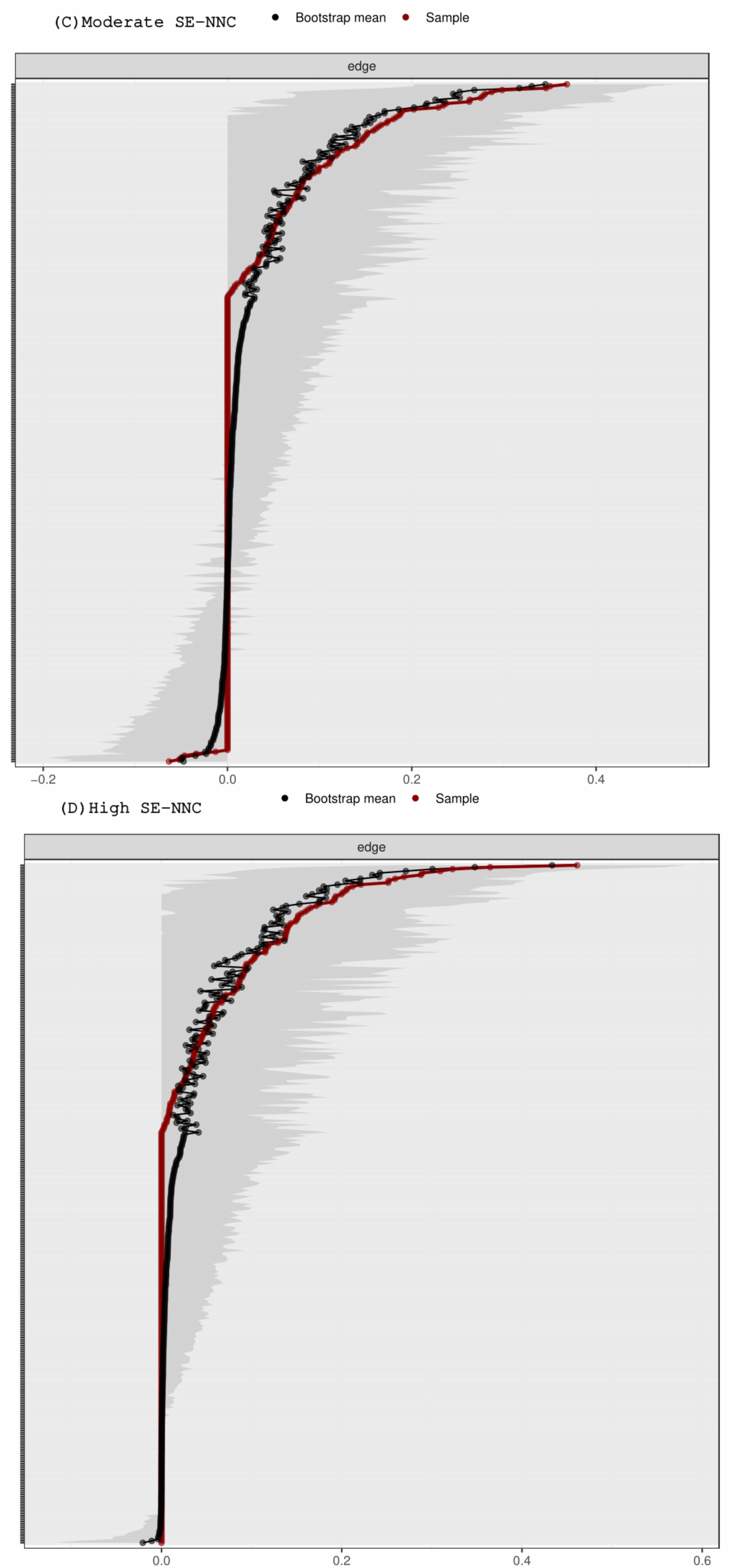


**Supplementary Figure 5** The 95% CIs of the edge weights in GGM network: (A) All people; (B) Low SE-NNC; (C) Moderate SE-NNC; (D) High SE-NNC.


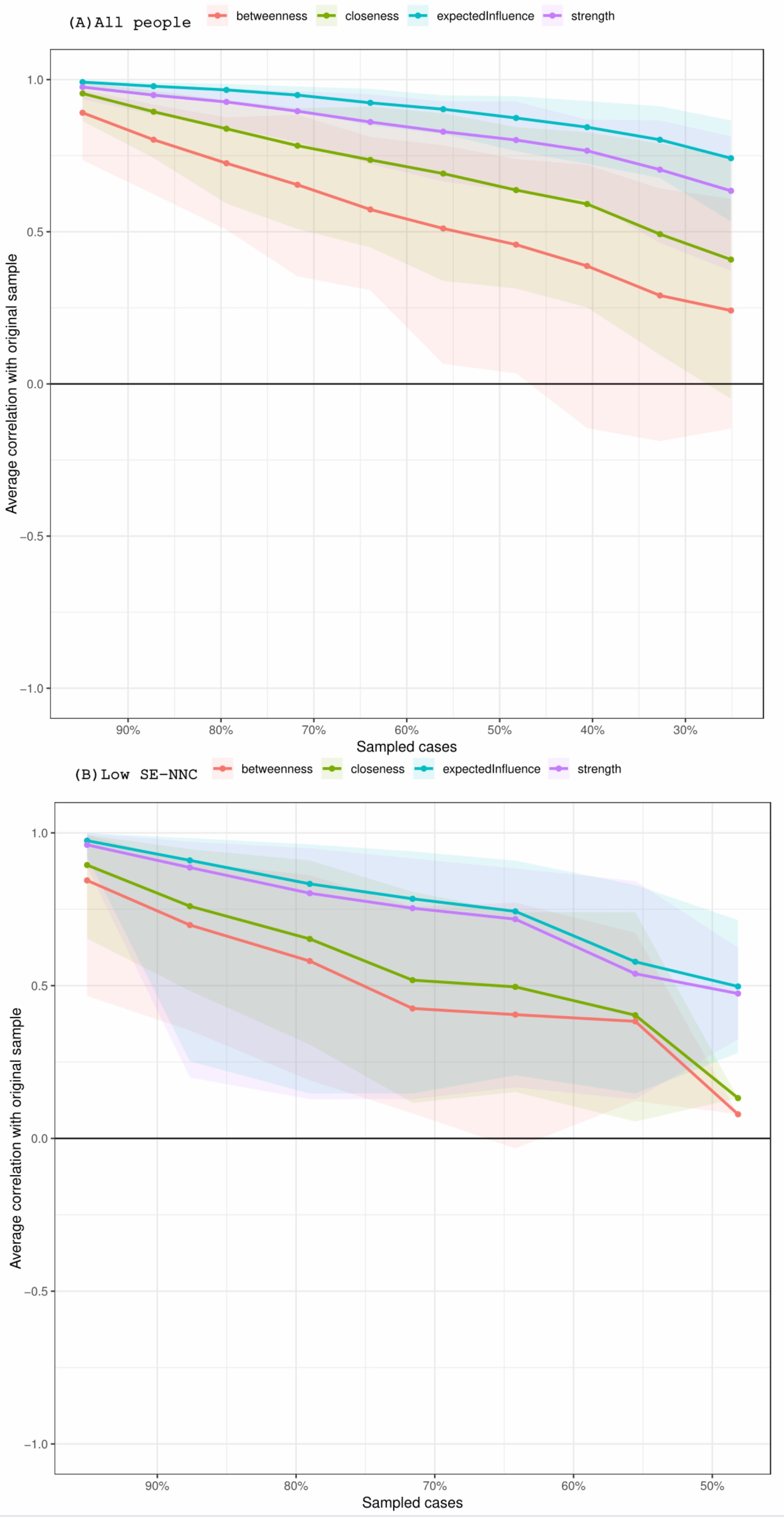


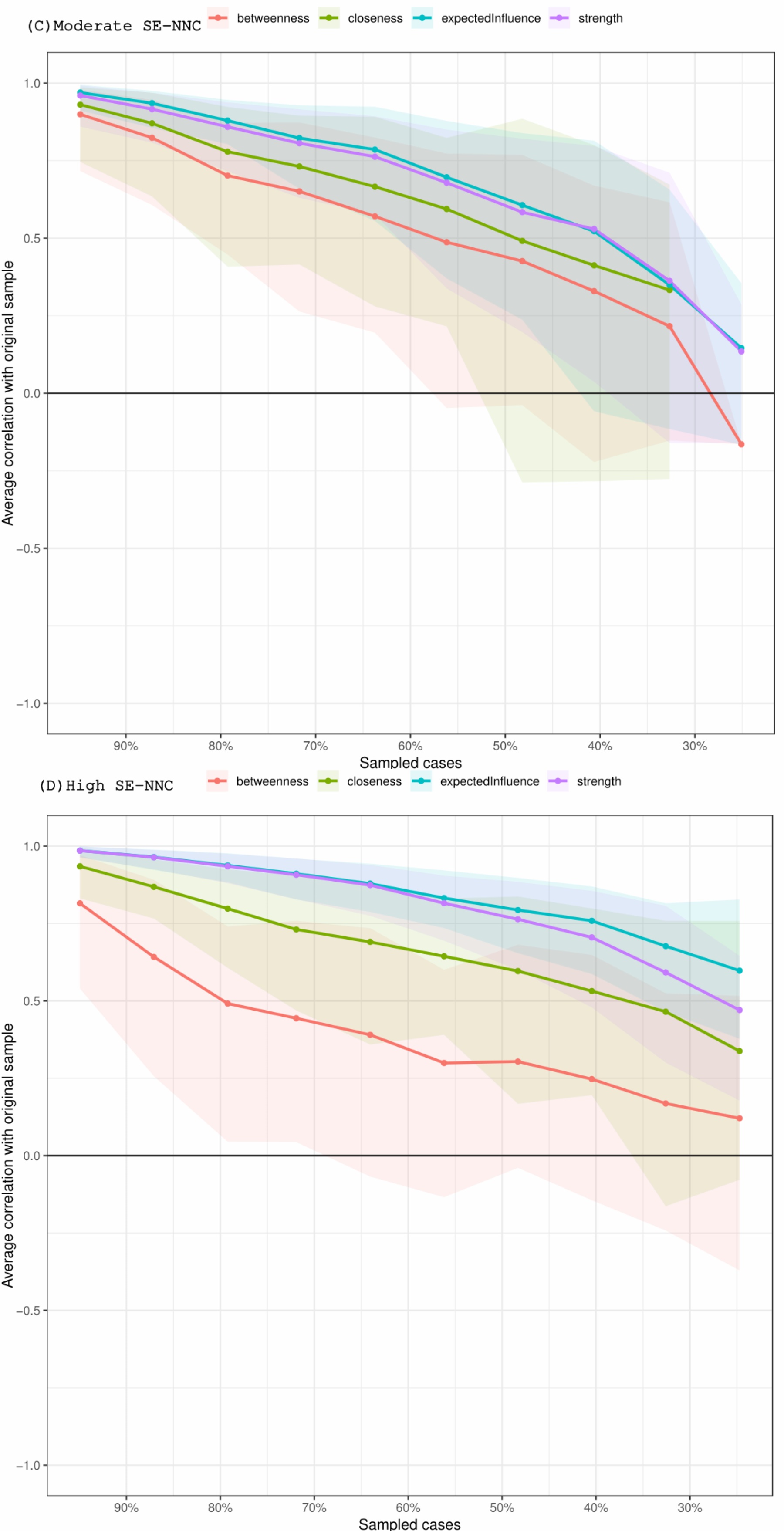


**Supplementary Figure 6** Stability test of 1000 bootstrap tests for centrality index in GGM Network: (A) All people; (B) Low SE-NNC; (C) Moderate SE-NNC; (D) High SE-NNC.
